# A Salivary Conglomerate with a Substantial Organic Component Mimicking a Mineral Sialolith in the Mandibular–Sublingual Gland Complex of a Dog

**DOI:** 10.3390/vetsci13101101

**Published:** 2026-10-16

**Authors:** Daniel Schidu, Vlad Zelinschi, Ozana-Maria Hrițcu, Ștefan Mihăiță Acomi, Raluca Adriana Ștefănescu, Eusebiu Viorel Șindilar, Liviu Cătălin Burtan, Gheorghe Solcan

**Affiliations:** 1 Clinics Department, Faculty of Veterinary Medicine, “Ion Ionescu de la Brad” Iasi University of Life Sciences, M. Sadoveanu Alley 8, 700489 Iasi, Romania; schidudaniel@gmail.com (D.S.); zelinschi.vlad96@gmail.com (V.Z.); stefan.acomi@iuls.ro (Ș.M.A.); raluca.stefanescu@iuls.ro (R.A.Ș.); eusebiu.sindilar@iuls.ro (E.V.Ș.); liviu.burtan@iuls.ro (L.C.B.); 2 Department of Preclinical Sciences, Faculty of Veterinary Medicine, “Ion Ionescu de la Brad” Iasi University of Life Sciences, M. Sadoveanu Alley 8, 700489 Iasi, Romania; ozana.hritcu@iuls.ro; 3 Academy of Agricultural and Forestry Sciences, 011646 Bucharest, Romania

**Keywords:** dog, mandibular salivary gland, monostomatic sublingual salivary gland, organic conglomerate, sialolith, FTIR spectrometry, sialoadenitis, sialoadenectomy

## Abstract

Solid lesions of the mandibular–sublingual salivary gland complex are rare in dogs. We report the case of an 11-year-old, 5 kg, intact female Maltese dog that presented with a non-painful, 2–3 cm left cervical swelling, soft at the surface and firm in depth. Fine-needle aspiration yielded clear, sero-mucous fluid. Radiographs revealed a well-defined, ovoid radiopaque structure measuring 3.1 × 2.9 cm in the cranial cervical region. Mandibular and monostomatic sublingual sialoadenectomy via a ventral approach revealed a large, heart-shaped, solid conglomerate within the glandular complex. Histopathological examination confirmed glandular atrophy, fibrosis and sialoadenitis. Fourier-transform infrared (FTIR) spectrometry of the submitted fragments indicated a substantial organic, protein- and lipid-rich component, with additional bands suggesting a possible minor mineral component. The dog healed completely, with no recurrence detected at the 2-week examination. Limitations include the absence of cross-sectional imaging and elemental analysis and the limited clinical follow-up. This case shows that protein-rich solid conglomerates should be included in the differential diagnosis of cervical swellings in dogs, that radiopacity alone does not establish mineral composition, and that complete surgical excision was associated with a favourable short-term outcome.

## Simple Summary

Solid lesions of the mandibular and sublingual salivary glands are rare in dogs and can be difficult to distinguish clinically from other cervical swellings. In this case report, we describe an 11-year-old Maltese dog with a large, well-defined, hard structure identified in the left cervical region on physical and radiographic examination. Surgical removal of the affected salivary gland complex retrieved a heart-shaped, solid conglomerate within the gland complex. Spectroscopic compositional analysis of the retrieved material suggested that it contained a substantial organic component rich in protein and fat (lipid), with a possible minor mineral component, whereas salivary stones are usually assumed to be mainly mineral. Microscopic examination of the surrounding tissue confirmed chronic inflammation, atrophy and scarring (fibrosis) of the gland. The dog recovered without complications and showed complete healing at two weeks after surgery. More advanced imaging, such as computed tomography, was not performed and could have helped to locate the lesion more precisely before surgery. This case illustrates that salivary lesions that appear on X-rays in dogs are not always mainly mineral, that protein-rich masses in the salivary gland should be considered in the diagnostic work-up of cervical swellings, and that surgical removal of the affected gland resulted in a favourable short-term outcome.

## 1. Introduction

Diseases of the salivary glands and ducts are uncommon overall in dogs and cats [1]. The mandibular gland lies between the linguofacial and maxillary veins, at the point where they unite to form the external jugular vein, and is fused to the monostomatic portion of the sublingual gland, with which it shares a closely related duct system—an anatomical relationship that explains why sialoadenectomy of this complex usually involves en bloc removal of both glands [1]. Salivary gland disorders in dogs include sialocele (salivary mucocele), sialoadenitis, sialolithiasis and neoplasia, with mucocele formation representing the most frequently reported condition and sialolithiasis considered rare in this species [2,3].

Sialoliths have been described most often in the parotid duct, whereas involvement of the mandibular–sublingual gland-duct complex is only occasionally reported, usually as isolated case reports [4,5,6,7]. Other non-obstructive parotid duct disorders, such as parotid duct ectasia, have also been described in dogs, further illustrating that salivary duct pathology in this species centres predominantly on the parotid gland rather than on the mandibular–sublingual complex [8]. Reported canine sialoliths are commonly composed of calcium carbonate or calcium phosphate, although non-mineral, protein-rich calculi have also been documented [7]. In humans, proteomic and spectroscopic studies have similarly shown that a substantial subset of sialoliths are predominantly organic or proteinaceous in composition, with mineral deposition occurring only secondarily, if at all, indicating that solid salivary-associated concretions cannot be assumed to be mineral in nature without compositional analysis [9].

Clinically, sialolithiasis of the mandibular–sublingual complex typically presents as a submandibular or cervical swelling, which may be difficult to distinguish from sialocele, abscess, foreign-body reaction or neoplasia on physical examination alone [3,5]. Plain radiography can demonstrate a mineral-opaque calculus and support the clinical suspicion, while definitive diagnosis relies on histopathological examination of the excised gland and duct [4,6]. Sialoadenectomy of the affected mandibular and sublingual glands, performed via either a ventral or lateral cervical approach, remains the standard treatment for sialocele and sialolithiasis of this gland complex, with a generally favourable long-term outcome reported in the literature [5,10,11].

We report a case of a large radiographically opaque conglomerate with a substantial organic, protein- and lipid-rich component associated with the mandibular–sublingual salivary gland complex, with secondary sialoadenitis, in a dog. The lesion was identified by clinical examination, cytology and cervical radiography, excised surgically via a ventral approach, and the surrounding glandular tissue was examined histopathologically, whereas the conglomerate itself was characterized by Fourier-transform infrared (FTIR) spectrometry. In this report, the term “conglomerate” is used for the excised lesion because its substantial organic component distinguishes it from a conventional, predominantly mineral sialolith; “sialolith” and “calculus” refer to previously reported salivary stones, and “concretion” is used as a general term encompassing both.

## 2. Case Presentation

### 2.1. Signalment

An 11-year-old, intact (non-spayed) female Maltese dog, weighing 5 kg, was presented to the surgery clinic.

### 2.2. History

The owners reported having first noticed the swelling accidentally, while petting the dog, approximately 3 weeks before presentation. They decided to seek veterinary care because the mass appeared to have increased markedly in size over the preceding 7 days. No changes in appetite or behaviour were noted by the owners, and the dog had not received any prior treatment for this condition.

### 2.3. Physical Examination

On presentation, the dog was alert, normothermic (rectal temperature 38.5 °C), with a respiratory rate of 30 breaths/min and a heart rate of 110 bpm. General physical status was unremarkable, and the dog maintained a normal appetite and water intake. A unilateral, non-painful mass, approximately 2–3 cm in diameter, was palpated in the left cervical/ventrolateral region, with mixed consistency—soft at the surface and firm in depth. Examination of the oral cavity and dentition revealed no abnormalities, and no changes were observed at the opening of the salivary duct. Regional lymph nodes (mandibular/retropharyngeal) were of normal size on palpation.

### 2.4. Preoperative Laboratory Findings

Preoperative complete blood count and serum biochemistry analyses were performed using a VetScan HM5 haematology analyser (Abaxis, Union City, CA, USA) and a BioSystems BA200 analyser (BioSystems, Barcelona, Spain), respectively, and are summarized in Table 1. Haematological reference intervals were those provided by the analyser manufacturer, and the biochemical reference intervals used were those of the Merck Veterinary Manual [12]. Mild increases in haemoglobin and MCH and a marked increase in alkaline phosphatase were noted, together with a mildly decreased ionized calcium concentration; the remaining parameters were within the reference intervals (Table 1).

### 2.5. Cardiological Assessment

Pre-anaesthetic cardiological evaluation, including auscultation, electrocardiography and transthoracic echocardiography (Vetus 8, Mindray, Shenzhen, China), revealed no abnormalities other than respiratory sinus arrhythmia and a wandering pacemaker, both considered physiological variants in dogs, and no contraindication to general anaesthesia was identified.

### 2.6. Diagnostic Imaging

**Figure 1:**
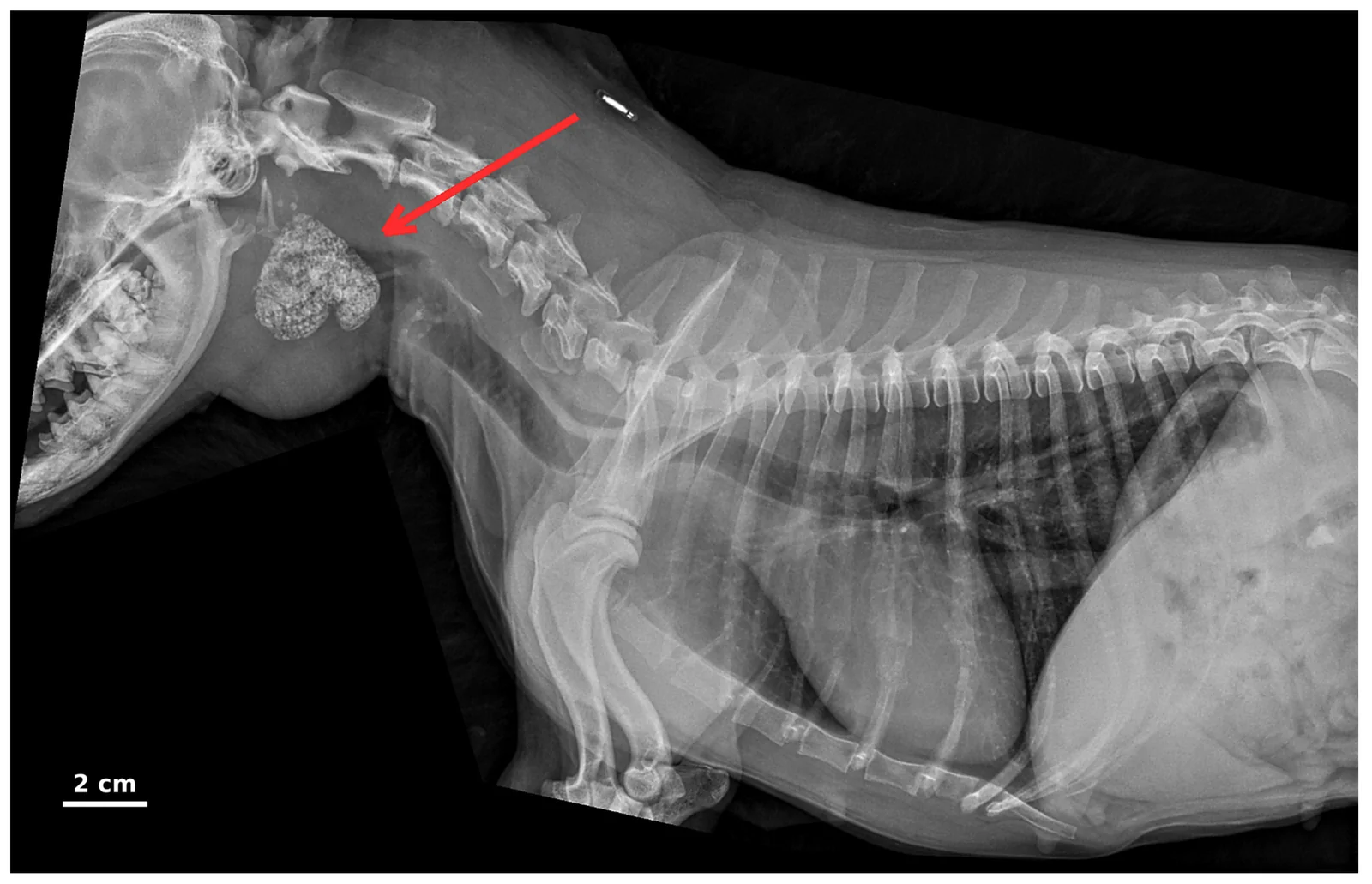
Right lateral radiographic view of the head, neck and thorax (composite of two overlapping digital exposures). The red arrow indicates a well-defined, approximately ovoid radiopaque structure (approximately 3.1 × 2.9 cm) in the cranial cervical region, subsequently identified as a conglomerate with a substantial organic, protein- and lipid-rich component; radiopacity alone did not establish its composition (see Section 2.12). Scale bar = 2 cm, derived from the digital measurement of the structure obtained with the radiography system software XVvue V1.0.0.82 (approximate, as radiographic magnification cannot be fully excluded).

Cervical and thoracic radiographs, including right and left lateral views, were obtained to evaluate the cervical mass using a Maxivet 400 DR digital veterinary radiography system (COMES ELECTRO S.r.l., Cavaria con Premezzo, Italy). The thoracic radiographs were obtained as part of pre-anaesthetic screening and to evaluate for possible pulmonary metastases, as neoplasia was included among the differential diagnoses. The right lateral view (Figure 1) showed the thoracic bony structures without traumatic or degenerative changes, a radiographically normal diaphragm, a trachea with sinuous course and normal calibre, and cardiovascular structures of normal radiographic appearance; the lungs showed no radiographic evidence of metastases. A well-defined, approximately ovoid radiopaque structure, measuring approximately 3.1 × 2.9 cm, was identified in the cranial cervical region, superimposed on the pharyngeal area on the lateral view, with possible lateralization; its exact localization could not be determined due to difficulty in patient restraint (the patient was anxious during the examination); for the same reason, an orthogonal ventrodorsal view, which would have helped to establish lateralization, could not be obtained. These findings raised suspicion of a salivary gland disorder associated with a solid, radiopaque structure, and a dental and surgical consultation was recommended.

### 2.7. Differential Diagnosis

Fine-needle aspiration of the soft, fluctuant component of the mass, performed on the day of presentation, one day before surgery, yielded clear, sero-mucous fluid. Cytological examination of the aspirated fluid revealed abundant amorphous mucinous material admixed with numerous foamy macrophages, occasional non-degenerate neutrophils, and variable numbers of erythrocytes; no cytological features of malignancy were identified in the aspirated fluid. Bacteriological examination of the aspirated fluid, submitted on the day of collection, performed at the microbiology laboratory of the Faculty of Veterinary Medicine, “Ion Ionescu de la Brad” Iași University of Life Sciences, included direct microscopic examination of a Gram-stained smear and culture on blood agar, MacConkey agar, brain heart infusion and chocolate agar, with incubation at 36 °C for 48 h; no bacterial growth was obtained; the excised tissue was not submitted for bacteriological culture. The cytological findings were consistent with salivary content, as found in a mucocele (sialocele); however, only the fluid component of the mass was sampled, and these findings were not necessarily representative of its solid component. Given the non-painful, slowly progressive cervical swelling with a mixed consistency, these cytological findings, and normal regional lymph nodes, several differential diagnoses were considered.

Sialocele (salivary mucocele) was initially considered because of the soft, fluctuant component and the mucous character of the aspirated fluid—zygomatic sialoceles and sialoceles located in the pharyngeal wall (pharyngeal sialoceles) have similarly been reported to present as slowly progressive, non-painful swellings in dogs [13,14]; however, the presence of a firm, well-defined radiopaque structure on radiographs was not typical of an uncomplicated mucocele and pointed instead towards a solid, obstructing conglomerate with secondary gland involvement. An abscess was considered less likely, although it could not be excluded on clinical grounds alone, as the mass was non-painful, the patient was afebrile and eupneic throughout the examination, and the complete blood count did not show a significant inflammatory leukogram. A foreign-body reaction was also considered given the firm component on palpation, but there was no history of trauma, oral/dental disease or foreign-body ingestion, and the well-circumscribed, ovoid radiopaque structure seen radiographically was considered more suggestive of a solid salivary-associated conglomerate than of a foreign body, although radiopacity alone did not establish its composition. Neoplasia was considered, given the patient’s age, but the absence of regional lymphadenopathy, the non-infiltrative, well-defined radiographic appearance of the mass, and the lack of pain on palpation made this differential less probable; however, as cytology had sampled only the fluid component, neoplasia could not be excluded before histopathological examination. Based on these findings, a solid obstructive conglomerate of the mandibular–sublingual gland complex with secondary sialoadenitis was considered the most likely working diagnosis, to be confirmed by surgical exploration, histopathological examination and compositional analysis.

### 2.8. Anaesthetic and Analgesic Protocol

Premedication consisted of methadone (Insistor, Richter Pharma AG, Wels, Austria) administered intravenously at 0.3 mg/kg (1.5 mg total dose for the 5 kg patient). General anaesthesia was induced with alfaxalone (Alfaxan, Jurox Pty Ltd., Rutherford, NSW, Australia), administered intravenously to effect (total dose 3 mg/kg), and maintained with isoflurane (IsoFlo, Zoetis Belgium SA, Louvain-la-Neuve, Belgium; 1–2%) in oxygen, with assisted (mechanical) ventilation using an R650 veterinary anaesthesia machine with an integrated ventilator (RWD Life Science Co., Ltd., Shenzhen, China); ketamine (Ketamidor, Richter Pharma AG, Wels, Austria) was administered as an intravenous bolus at 2 mg/kg during the maintenance phase for perioperative analgesia. Additional analgesia was provided by local infiltration of lidocaine (LidoBel 20 mg/mL, Bela-Pharm GmbH & Co. KG, Vechta, Germany) at multiple points along the planned incision line, at a dose of 4 mg/kg (20 mg total dose; 1 mL for the 5 kg patient). Intraoperative fluid therapy consisted of a balanced crystalloid solution (Ringer’s solution; Infomed Fluids SRL, Bucharest, Romania) administered at a constant rate of 5 mL/kg/h using an infusion pump (BeneFusion VP3 Vet, Mindray, Shenzhen, China), with fluids warmed by a blood and infusion warmer (Eickemeyer, Tuttlingen, Germany). The duration of surgery was 90 min. Intraoperative monitoring was performed with a multiparameter veterinary monitor (uMEC 10 Vet, Mindray, Shenzhen, China) and included electrocardiography, heart rate, pulse oximetry (SpO_2_), capnography (end-tidal CO_2_, EtCO_2_), non-invasive (oscillometric) blood pressure, respiratory rate and body temperature. Heart rate ranged from 90 to 120 bpm, SpO_2_ remained between 96% and 98%, EtCO_2_ was maintained at approximately 40 mmHg with a respiratory rate of approximately 25 breaths/min, and mean arterial pressure was maintained at approximately 70 mmHg, with no episodes of hypotension. Body temperature was 38.5 °C at induction and 38.1 °C at the end of surgery; normothermia was supported with a forced-warm-air warming system (WMS-1501, AEOLUS International Pet Products, Irving, TX, USA).

### 2.9. Surgical Technique

**Figure 2:**
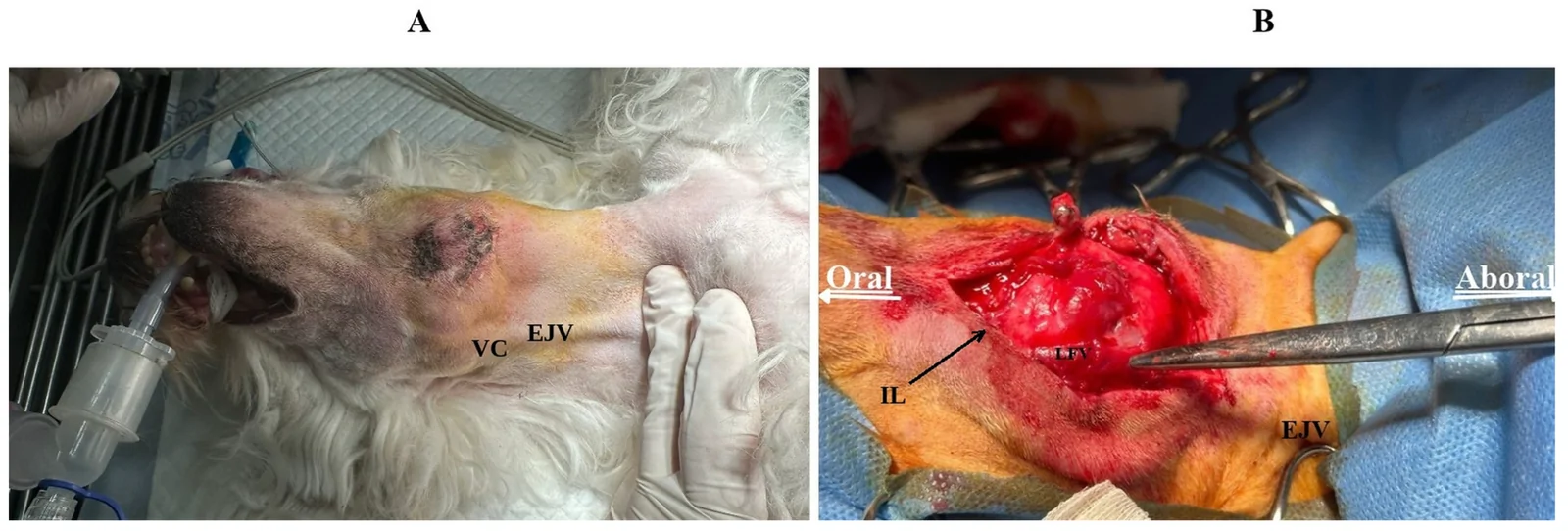
(**A**) Dorsal recumbency positioning of the patient prior to surgery. EJV: external jugular vein; VC: venous confluence of the linguofacial and maxillary veins. (**B**) Intraoperative view showing the paramedian skin incision line (IL), the external jugular vein (EJV) and the linguofacial vein (LFV). Oral: oral direction; Aboral: aboral direction.

**Figure 3:**
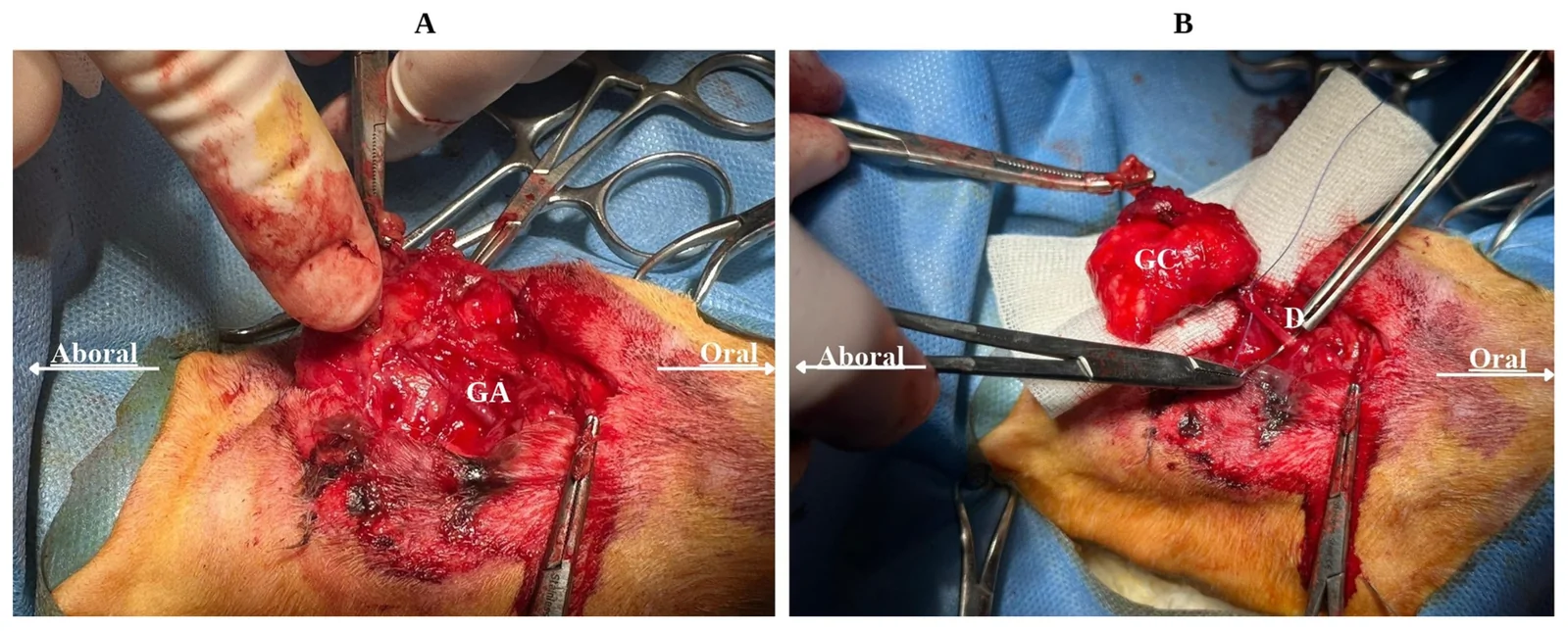
(**A**) Dissection of the glandular complex, exposing the glandular artery (GA), a branch of the facial artery. (**B**) Ligation of the duct D at the level of the glandular complex (GC). Oral: oral direction; Aboral: aboral direction.

**Figure 4:**
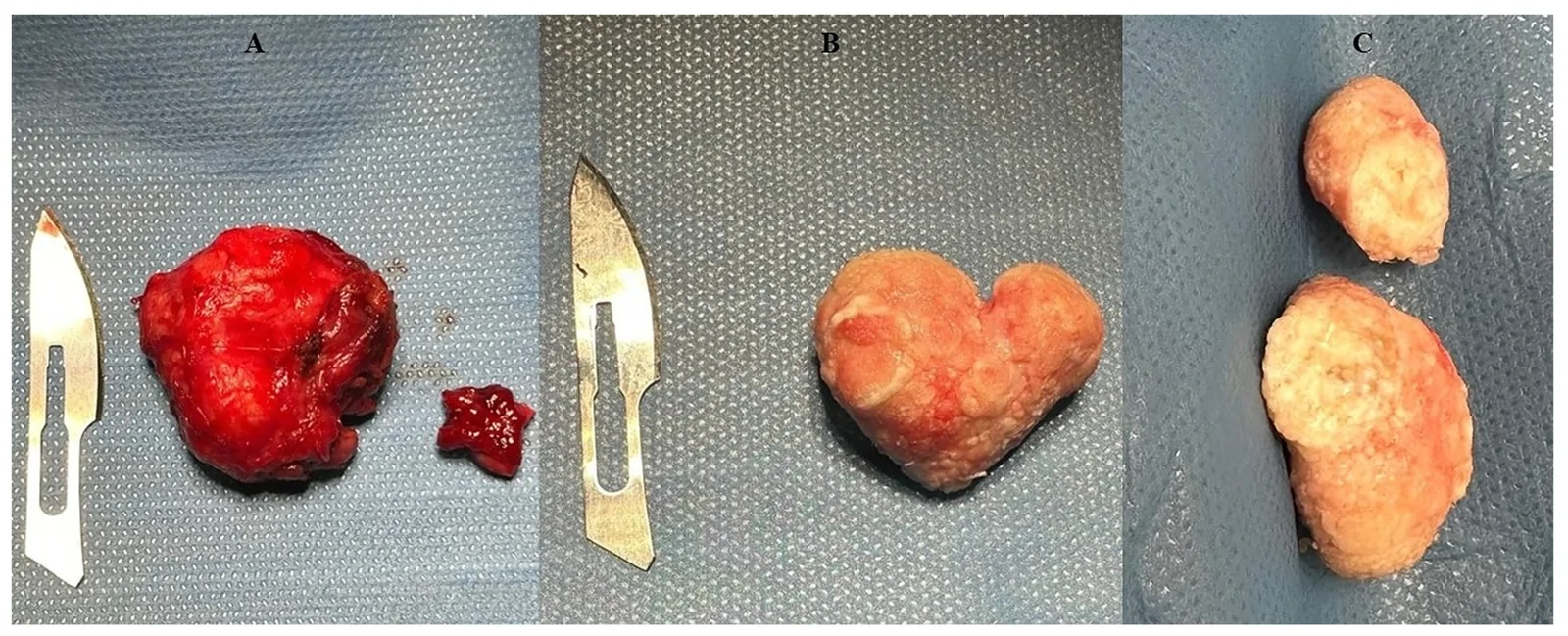
Gross appearance of the excised glandular complex and of the conglomerate; a scalpel blade is included in (**A**,**B**) as a size reference. (**A**) Complex still enclosed within its capsule. (**B**) Heart-shaped conglomerate after removal of the surrounding capsule. (**C**) Appearance of the conglomerate after sectioning.

A ventral paramedian approach was selected to expose and excise the affected mandibular–monostomatic sublingual salivary gland complex. This approach provides direct access to the mandibular and monostomatic sublingual salivary glands and has been associated with a lower risk of recurrence than the lateral approach in dogs undergoing sialoadenectomy for sialocele, although wound-related complications may be more frequent [15]. The patient was positioned in dorsal recumbency (Figure 2A). A 3–4 cm paramedian skin incision was made along the longitudinal axis of the swelling, approximately 10 mm from the external jugular vein. The linguofacial vein was identified near its union with the maxillary vein to form the external jugular vein (Figure 2B). The linguofacial vein was closely apposed to and partially adherent to the capsule of the mandibular salivary gland; it was carefully dissected free while preserving its integrity. The mandibular salivary gland and monostomatic sublingual salivary gland were exposed as a single glandular complex. The glandular branch of the facial artery was identified and ligated (Figure 3A). Dissection was continued until the mandibular and sublingual ducts were identified; the ducts were dissected for a short distance from the glandular complex and then ligated with 3-0 absorbable monofilament suture material and transected close to the gland (Figure 3B). The glandular complex was then removed en bloc through the skin incision, aided by gentle stretching of the skin edges. Its gross appearance, still enclosed within its capsule, is shown in Figure 4A. Following removal of the capsule, a heart-shaped, solid conglomerate was retrieved from within the glandular complex (Figure 4B); the conglomerate was located within the glandular complex and was not found within the lumen of the mandibular or sublingual duct; its appearance after sectioning is shown in Figure 4C. After sectioning, the conglomerate was divided into a fragment of approximately 2 × 1.5 cm, which was submitted for histopathological examination together with the glandular tissue (Section 2.11), and the remaining material, which was submitted for FTIR spectrometric analysis (Section 2.12). The surgical site was closed in two layers using 3-0 absorbable monofilament suture material: the subcutaneous tissue with a continuous pattern and the skin with simple interrupted sutures.

### 2.10. Perioperative and Postoperative Medication

Postoperative anti-inflammatory and analgesic treatment consisted of meloxicam (Melovem injectable, Dopharma Research B.V., Raamsdonksveer, The Netherlands), administered subcutaneously at 0.2 mg/kg immediately after surgery, followed by an oral suspension of meloxicam (Rheumocam, Chanelle Pharmaceuticals Manufacturing Ltd., Loughrea, Ireland) at 0.1 mg/kg once daily for a further 4 days. Antibiotic therapy consisted of amoxicillin-clavulanate (Kesium, Ceva Santé Animale, Libourne, France), administered orally at 12.5 mg/kg every 12 h for 5 days. This treatment was initiated empirically, before the bacteriological result was available (the 48-h culture of the aspirate collected one day before surgery was still pending), because of the suspected secondary sialoadenitis and the surgical exposure of a ductal system communicating with the oral cavity, and was completed as a short course. The surgical dressing was changed at 24 h postoperatively.

### 2.11. Gross and Histopathological Examination

**Figure 5:**
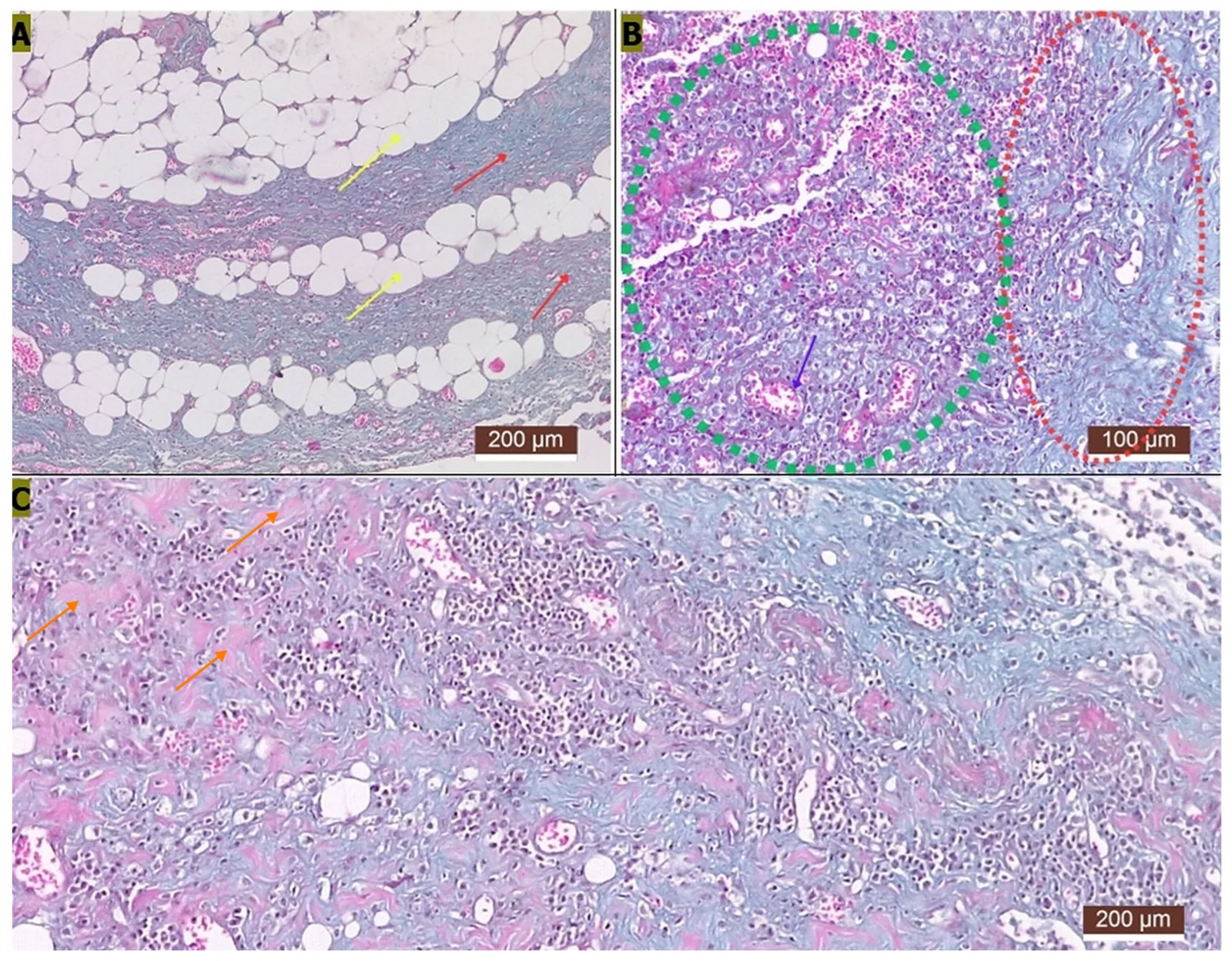
Histological appearance of the excised glandular complex (Masson’s trichrome stain). (**A**) Yellow arrows: adipose tissue infiltration; red arrows: fibrotic tissue. (**B**) Green outline: atrophic secretory acini with moderate inflammatory infiltrate and areas of haemorrhage; purple arrow: newly formed vessels; red outline: fibrosis. (**C**) Orange arrows: areas consistent with mineralization of the glandular parenchyma (not confirmed by a mineral-specific stain). Scale bars: 200 µm (**A**,**C**) and 100 µm (**B**).

Histopathological examination was performed on the excised glandular complex, fixed in 10% formalin; one section stained with Masson’s trichrome was examined, and routine haematoxylin and eosin (H&E) staining was not performed (Figure 5). A fragment of the conglomerate (approximately 2 × 1.5 cm), white in colour, with a rough surface and hard consistency, was submitted together with the glandular tissue but was not sectioned; only the glandular tissue was processed and examined histologically, and no mineral-specific stain (e.g., von Kossa or Alizarin red) was performed. The surrounding tissue, consistent with residual salivary gland parenchyma and/or excretory duct, contained occasional groups of secretory-type epithelial cells with foamy cytoplasm and centrally located nuclei, together with cells showing basophilic, granular cytoplasm, possibly originating from a mixed salivary acinus. Fibrous connective tissue predominated, with numerous newly formed vessels giving a granulation-tissue-like appearance in some areas (Figure 5A). A mixed inflammatory infiltrate and areas of haemorrhage (Figure 5B), as well as areas consistent with mineralization (Figure 5C), were also observed. Adipose tissue infiltration was also present within the fibrous stroma (Figure 5A). The excised specimen included a lymph node with a largely preserved follicular architecture, mildly rarefied predominantly in the cortical zone, showing haemosiderosis and lipofuscinosis. The histopathological diagnosis was salivary gland atrophy, fibrosis, and sialoadenitis.

### 2.12. Fourier-Transform Infrared (FTIR) Spectrometric Analysis of the Conglomerate

**Figure 6:**
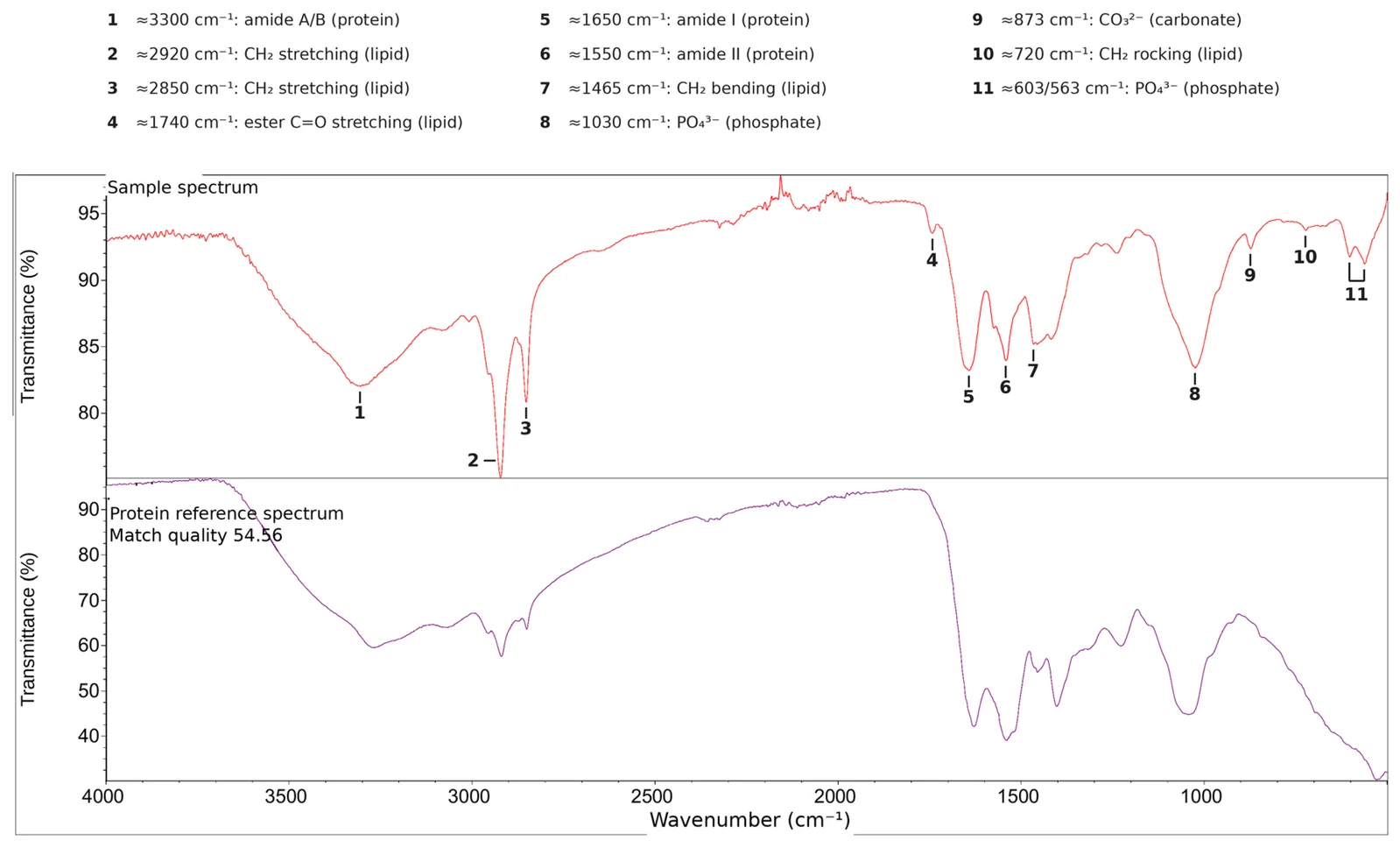
Fourier-transform infrared (FTIR) spectrum of the excised conglomerate (analysis performed by Laboklin GmbH & Co. KG, Bad Kissingen, Germany). (**Upper**) trace (red): sample spectrum. (**Lower**) trace (purple): closest reference spectral match, classified as protein, with a match quality of 54.56. Both traces plot transmittance (%) against wavenumber (cm^−1^). Numbered bands of the sample spectrum and their tentative assignments are given in the key above the spectra: amide A/B (1), amide I (5) and amide II (6) bands of protein; CH_2_ stretching (2, 3), ester C=O stretching (4), CH_2_ bending (7) and CH_2_ rocking (10) bands of long-chain lipid; and phosphate (8, 11) and carbonate (9) bands of an apatite-like mineral phase. The annotations of the original laboratory output were translated from German into English; the spectral traces are unmodified, except for a short segment of the reference trace previously obscured by the original annotation; the band numbers and the key were added by the authors.

The remaining material of the excised conglomerate (Section 2.9) was submitted to an external accredited diagnostic laboratory (Laboklin GmbH & Co. KG, Bad Kissingen, Germany) for compositional analysis by Fourier-transform infrared (FTIR) spectrometry. The analysis was performed within the laboratory’s accredited scope for stone analysis, which includes salivary calculi of all animal species. The submitted material, as described in the laboratory report, comprised nine beige fragments with a rough surface, ranging in size from approximately 1 × 1 mm to 22 × 22 mm. The material was white when retrieved at surgery and had become beige by the time of analysis, probably as a result of dehydration, which may also have reduced the size of the fragments. The FTIR spectrum of the sample (Figure 6) was compared against the laboratory’s reference spectral library using automated library-search software Opus version 8.5; the closest match was to a protein reference spectrum, with a reported match quality of 54.56. This value represents the similarity index generated by the laboratory’s library-search software, with higher values indicating closer agreement between the sample and reference spectra; a value of 54.56 falls below the acceptance criteria used for FTIR library identification of uroliths. Acceptance criteria are software- and laboratory-specific; in a published protocol for canine urolith analysis, hit-quality values of at least 800 on a 0–1000 scale are considered high-confidence and values of 700–800 tentative [16]. The laboratory did not report the scale or acceptance criterion applied, but a value of 54.56 falls below these criteria whether expressed on a 0–100 or a 0–1000 scale. Both the sample spectrum and the reference spectrum showed the broad amide A/B absorption band near 3300–3000 cm^−1^ and the characteristic amide I and amide II bands near 1650 cm^−1^ and 1550 cm^−1^, consistent with peptide backbone vibrations of a proteinaceous matrix [17]. In addition to the amide bands, the sample spectrum showed further absorption bands at approximately 2920/2850, 1740, 1465 and 720 cm^−1^, compatible with long-chain lipid (CH_2_ and ester carbonyl) vibrations [17,18], and at approximately 1030, 873 and 603/563 cm^−1^, compatible with phosphate and carbonate vibrations of an apatite-like mineral phase [19]; these bands were absent from, or weak in, the protein reference spectrum. In the laboratory report, proteins were listed as the main constituent of the analysed material, and no minor or mineral constituent was listed; however, this reflects the automated single-component library classification rather than a quantitative measurement. On the basis of these findings, the analysed material was considered to contain a substantial organic, protein- and lipid-rich component, with spectral features suggesting a possible minor mineral component. Because FTIR band intensities do not quantify the relative proportions of these constituents, the proportions of protein, lipid and mineral in the analysed material could not be determined.

### 2.13. Postoperative Course and Follow-Up

The patient was discharged 24 h after surgery. Voluntary food and water intake resumed 6 h postoperatively. A surgical recheck was performed at 48 h, at which time the dressing was changed again. Skin sutures were removed at 2 weeks postoperatively. Complete wound healing was achieved, with no evidence of recurrence at the 2-week examination. No further clinical re-examination was performed after suture removal. At telephone follow-up 4 months postoperatively, the owner reported no recurrence of the cervical swelling, complete healing of the surgical wound and a good general condition of the dog, and was satisfied with its quality of life; the owner considered a further clinical re-examination unnecessary.

## 3. Discussion

Solid lesions of the mandibular–sublingual gland-duct complex are uncommon in dogs, and most reported canine sialoliths originate in the parotid duct; involvement of the mandibular gland has been documented mainly through isolated case reports [4,6,7]. Concurrent sialoadenitis, as observed in the present case, has been described in association with ductal obstruction by a solid concretion, which promotes secondary parenchymal inflammation [3,5].

A key finding of the present case was the marked discordance between the radiographic opacity of the lesion and its biochemical composition. FTIR spectrometry of the submitted fragments indicated a substantial organic, protein- and lipid-rich component, with additional bands suggesting a possible minor mineral component, indicating that the excised structure differed from a conventional, predominantly mineral sialolith. This finding is consistent with a growing body of evidence that salivary calculi are not uniformly mineral in composition. In human medicine, organic and mixed sialoliths are well recognized, and complex organic matrices have been identified even in radiographically dense calculi and in concretions at other sites [9,20,21,22,23]; whether these observations apply to canine salivary concretions remains unknown. The marked radiographic opacity of a conglomerate with such a substantial organic component requires explanation. The additional spectral bands compatible with a phosphate/carbonate (apatite-like) phase suggest that a minor mineral fraction, below the threshold for automated library classification or unevenly distributed within the conglomerate, was present and may also account for the only moderate library match quality. Such a minor mineral fraction, possibly combined with dense compaction and dehydration of the organic matrix, may explain the radiographic opacity. The focal mineralization observed histopathologically was located in the surrounding glandular tissue, not in the conglomerate, and therefore does not inform the composition of the conglomerate itself. Radiopaque sialoliths reported as non-mineral have previously been described in dogs [7]. In the absence of elemental analysis, however, this interpretation remains hypothetical.

Because the conglomerate was located within the glandular complex rather than within the duct lumen, its classification as a ductal concretion could not be established. An alternative interpretation is a sialocele in which inspissated salivary secretion, enclosed by a pseudocapsule of granulation tissue and fibrous tissue, underwent dystrophic mineralization; secondary changes such as osseous metaplasia have been described within salivary mucoceles in dogs [3]. The mucinous cytology of the aspirated fluid, the soft, fluctuant component of the mass and the granulation tissue and fibrosis observed histopathologically would be compatible with this interpretation. The available findings did not allow these two possibilities to be distinguished. The term “conglomerate” is therefore used descriptively, without implying a ductal origin.

The pathogenesis of salivary concretions has not been fully elucidated, and the mechanisms discussed here are hypotheses derived largely from human studies rather than mechanisms demonstrated in the present dog. Salivary stasis or reduced salivary flow is considered a central predisposing factor, promoting the accumulation of secretory material within the duct system [24,25]. According to the organic-nidus and retrograde theories, mucous plugs, glycoproteins, desquamated epithelial cells and inflammatory debris, together with bacteria ascending from the oral cavity, may form an initial organic core, around which further organic and, variably, inorganic material is deposited [25,26]. Secondary inflammation may subsequently lead to fibrosis and further ductal narrowing, potentially establishing a self-perpetuating cycle of stasis, inflammation and concretion growth [25]. In the present dog, total calcium and phosphorus were within the reference intervals, and ionized calcium was mildly decreased, which does not support a hypercalcaemic contribution; the increased alkaline phosphatase activity was not further investigated, and an underlying systemic condition therefore cannot be excluded. These hypothetical mechanisms would be compatible with the glandular atrophy, fibrosis and sialoadenitis identified histopathologically, but they could not be demonstrated in the present case, and the initiating event remains unknown.

A particularly notable feature of the present case is the large size of the conglomerate, measuring approximately 3.1 × 2.9 cm on radiographic and gross examination—considerably larger than the canine sialoliths cited in this report, such as the 4 mm calculus described by Ryan et al. [6] or the 11 × 2.2 mm mineral-attenuating structure identified by Coutin et al. [4]. In human medicine, submandibular calculi exceeding 3 cm are considered rare and are classified as “giant” sialoliths, a threshold historically defined for mineral calculi [27]; the maximum dimension of the present conglomerate (3.1 cm) slightly exceeds this threshold, although the threshold was defined for human mineral calculi and its applicability to a concretion with a substantial organic component in a dog is uncertain.

Mandibular–sublingual sialoadenectomy via a ventral or lateral cervical approach is considered the definitive treatment for sialocele and sialolithiasis involving this gland complex, and complete excision of the gland and duct is associated with a low recurrence rate regardless of the approach chosen [10,11,28].

In the present case, complete surgical excision of the mandibular and sublingual glandular complex via a ventral approach resulted in an uneventful recovery, with resumption of voluntary food and water intake within 6 h after surgery and complete wound healing without evidence of recurrence at the time of suture removal, two weeks postoperatively; this favourable short-term outcome is consistent with the results reported after sialoadenectomy of this gland complex [5,11].

This report has several limitations. First, there was the absence of advanced cross-sectional imaging, such as computed tomography or sialography, which could have provided additional preoperative information on the precise ductal or glandular localization of the conglomerate, as described in other reports [4]. Furthermore, only lateral radiographic views were obtained; an orthogonal ventrodorsal view could not be acquired because of the patient’s anxiety and difficult restraint, which limited the radiographic assessment of the lateralization and exact location of the opacity (Section 2.6). In addition, clinical follow-up was limited to the immediate postoperative period and to suture removal at two weeks; longer-term follow-up (4 months) was based solely on owner report by telephone, without clinical or imaging reassessment, and extended clinical follow-up would be required to more definitively exclude late recurrence. Four further limitations relate to the compositional analysis. First, FTIR spectrometry was performed on nine submitted fragments rather than on the entire gross specimen; therefore, the compositional findings apply specifically to the analysed material and cannot fully exclude compositional heterogeneity in unsampled portions, particularly given that histopathology identified focal mineralization within the surrounding glandular tissue. Second, instrument-level details, including the sampling technique, sample preparation, handling of the individual fragments and the identity of the reference library, were not reported by the external laboratory. Third, FTIR spectrometry classifies the general biochemical class of the dominant constituent based on characteristic amide absorption bands but does not resolve the identity of the specific protein(s) present; the exact protein(s) forming the conglomerate were not further characterized in this case, and techniques such as mass-spectrometry-based proteomics would be required to establish this. Fourth, the spectral match quality reported by the laboratory (54.56) was below the acceptance criteria used for FTIR library identification [16]. Because library searching compares the sample spectrum with single-component reference spectra, a mixed composition, as suggested by the additional lipid- and phosphate/carbonate-related absorption bands, is a likely contributor to this moderate match; the result should therefore be interpreted as identifying the dominant biochemical class of the analysed material rather than as an absolute quantification of its composition. Finally, the histopathological examination was limited. The diagnosis was based on a single section stained with Masson’s trichrome, without haematoxylin and eosin staining. The conglomerate fragment itself was not sectioned, so its microscopic structure could not be assessed. The areas of mineralization were identified on Masson’s trichrome alone, which is not specific for mineral, without confirmation by a mineral-specific stain such as von Kossa or Alizarin red. Orthogonal techniques, such as energy-dispersive X-ray spectroscopy or X-ray diffraction, were not performed and would be required to confirm or exclude a mineral component; their use is recommended in future cases of this type.

From a clinical perspective, this case suggests that a large, well-defined radiopaque structure associated with the mandibular–sublingual gland complex should prompt consideration of an obstructive salivary concretion in the differential diagnosis of cervical swellings in dogs, even when the oral cavity and salivary duct opening appear normal; that radiographic opacity alone does not allow the composition of such a concretion to be inferred; and that compositional analysis of the retrieved material, ideally combining spectroscopic and elemental techniques, should be considered to improve the characterization of these uncommon lesions.

## 4. Conclusions

This case describes a large radiographically opaque conglomerate with a substantial organic, protein- and lipid-rich component associated with the mandibular–sublingual salivary gland complex in a dog and accompanied by chronic salivary gland atrophy, fibrosis and sialoadenitis. FTIR spectrometry of the submitted material indicated a substantial protein- and lipid-rich organic component, with spectral features suggesting a possible minor mineral component, indicating that radiopacity alone does not allow the composition of a salivary concretion to be inferred. Surgical excision of the affected mandibular–monostomatic sublingual gland complex resulted in complete clinical resolution, with no recurrence detected at the 2-week examination and none reported by the owner at 4 months. Compositional analysis should be considered whenever solid salivary-associated lesions that appear radiopaque on radiographs are recovered, particularly when their gross, radiographic and histopathological features do not unequivocally establish a mineral calculus.

## Figures and Tables

**Table 1:** Preoperative laboratory findings.

Parameter	Unit	Result	Reference Interval
Complete blood count (CBC)			
WBC	10^9^/L	13.57	6–17
Lymphocytes (LYM)	10^9^/L	2.19	1–4.8
Monocytes (MON)	10^9^/L	0.72	0.2–1.5
Neutrophils (NEU)	10^9^/L	10.1	3–12
Eosinophils (EOS)	10^9^/L	0.36	0–0.8
Basophils (BAS)	10^9^/L	0.2	0–0.4
RBC	10^12^/L	7.5	5.5–8.5
Haemoglobin (HGB)	g/dL	18.8 ↑	12–18
Haematocrit (HCT)	%	54.01	37–55
MCV	fL	72	60–77
MCH	pg	25.1 ↑	19.5–24.5
MCHC	g/dL	34.8	31–39
RDWc	%	18.6	14–20
Platelets (PLT)	10^9^/L	450	165–500
MPV	fL	11	3.9–11.1
Serum biochemistry			
AST/GOT	U/L	32	14–45
Alkaline phosphatase (ALP)	U/L	283 ↑	1–114
ALT/GPT	U/L	36	10–109
Phosphorus	mg/dL	4.42	2.9–5.3
Total protein	g/L	66	54–75
γGT	U/L	2	1–9.7
Calcium	mg/dL	9.14	9.1–11.7
Magnesium	mg/dL	1.66	1.6–2.4
Iron	µg/dL	51.2	33.0–147.0
Albumin	g/L	32	28–39
Total bilirubin	mg/dL	0.06	0.0–0.4
Creatine kinase (CK)	U/L	48	36–155
Triglycerides	mg/dL	126	<150
Cholesterol	mg/dL	181	135–278
Potassium	mmol/L	4.76	3.8–5.6
Sodium	mmol/L	141	140–154
Ionized calcium	mmol/L	1.20 ↓	1.25–1.45

Reference intervals: haematology, manufacturer-provided (VetScan HM5, Abaxis); serum biochemistry, Merck Veterinary Manual [12]. ↑ = values increased in comparison with reference; ↓ = values decreased in comparison with reference.

## Data Availability

The original contributions presented in this study are included in the article. Further inquiries can be directed to the corresponding author.

## Abbreviations

The following abbreviations are used in this manuscript:

CBC	Complete blood count
WBC	White blood cells
RBC	Red blood cells
MCV	Mean corpuscular volume
MCH	Mean corpuscular haemoglobin
MCHC	Mean corpuscular haemoglobin concentration
RDWc	Red cell distribution width, coefficient of variation
PLT	Platelets
MPV	Mean platelet volume
AST/GOT	Aspartate aminotransferase
ALT/GPT	Alanine aminotransferase
ALP	Alkaline phosphatase
CK	Creatine kinase
EJV	External jugular vein
LFV	Linguofacial vein
IL	Incision line
VC	Venous confluence (linguofacial and maxillary veins)
FTIR	Fourier-transform infrared (spectrometry)
GC	Glandular complex
GA	Glandular artery
